# Eosinophil-to-Lymphocyte Ratio as a Potential Inflammatory Marker Associated with Endometrial Cancer: An Analysis of NHANES 2017–2023 and an Independent Institutional Cohort

**DOI:** 10.3390/diagnostics16213675

**Published:** 2026-11-06

**Authors:** Hanna Moon, Bu Yong Kim, Sunghoon Kim

**Affiliations:** Department of Obstetrics & Gynecology, Yonsei University Health System, Seoul 03722, Republic of Korea; hmoon@yuhs.ac (H.M.);

**Keywords:** Eosinophil-to-Lymphocyte Ratio (ELR), uterine cancer, endometrial cancer, systemic inflammatory markers, biomarkers, NHANES

## Abstract

**Background/Objectives**: This study evaluates the Eosinophil-to-Lymphocyte Ratio (ELR) as a systemic inflammatory marker associated with endometrial cancer using a two-stage approach combining a national population-based discovery analysis of self-reported uterine cancer (NHANES) and an independent institutional cohort of histologically confirmed endometrial cancer. **Methods**: In the discovery cohort, survey-weighted logistic regression was conducted among 6859 women from NHANES 2017–2023, comparing 6757 non-cancer controls with 102 women with self-reported gynecological cancers (45 cervical, 38 uterine, and 19 ovarian); the uterine cancer model was adjusted for age and BMI. In the independent institutional cohort, 901 patients with histologically confirmed primary endometrial cancer and 200 benign surgical controls were analyzed using the Mann–Whitney U test, unadjusted logistic regression, and Receiver Operating Characteristic (ROC) curve analysis. Adjusted analyses were not performed in this cohort. **Results**: In NHANES, mean ELR was higher in the uterine cancer group than in controls (0.1204 vs. 0.0868, *p* = 0.0193). After adjustment for age and BMI, ELR was associated with higher odds of self-reported uterine cancer (OR = 10.288 per 1.0-unit increase, 95% CI: 1.722–61.472, *p* = 0.0130; equivalent to OR = 1.26 per 0.1-unit increase, 95% CI: 1.06–1.51). In the institutional cohort, median ELR was significantly higher in endometrial cancer patients than in benign controls (0.1180 [IQR: 0.1380] vs. 0.0487 [IQR: 0.0574], *p* < 0.001). Unadjusted logistic regression showed that higher ELR was associated with endometrial cancer (OR = 2.725 per 0.1-unit increase, 95% CI: 2.154–3.515, *p* < 0.001). ROC analysis showed moderate discriminative capability (AUC = 0.7147, 95% CI: 0.6780–0.7514), with an exploratory (non-validated) cut-off of 0.0810 (sensitivity: 65.5%; specificity: 74.8%). **Conclusions**: Higher ELR was associated with self-reported uterine cancer in NHANES after adjustment for age and BMI, and with histologically confirmed endometrial cancer in an unadjusted institutional analysis, with moderate discriminative performance. These hypothesis-generating findings do not establish an independent association; ELR may be a candidate complementary, non-invasive marker, but its clinical utility requires confirmation in adjusted, prospective studies.

## 1. Introduction

The interplay between the immune system and oncogenesis is a cornerstone of modern cancer research, particularly regarding how systemic inflammatory responses dictate disease progression [1]. Unlike acute inflammation, which is a self-limiting protective response, chronic inflammation creates a persistent pro-tumorigenic microenvironment [1]. This state fosters genomic instability through the production of Reactive Oxygen Species (ROS) and Reactive Nitrogen Species (RNS), promotes angiogenesis, and suppresses effective anti-tumor immune responses.

Cancer and autoimmune diseases share dysregulated immune surveillance and chronic inflammatory signaling, including oxidative stress, cytokine-driven pathways such as IL-6 and TNF-α signaling through STAT3 and NF-κB, impaired surveillance under immunosuppressive therapy, leukocyte-derived growth factors such as VEGF, and immune checkpoint pathways such as PD-1/PD-L1 [2,3,4,5,6,7,8,9,10]. The clinical adoption of immune checkpoint inhibitors in gynecological oncology [11,12] underscores the need for accessible systemic markers that capture the underlying immune-inflammatory equilibrium.

Historically, various white blood cell (WBC) ratios have been used to gauge this balance. The Neutrophil-to-Lymphocyte Ratio (NLR) is the most extensively documented; here, elevated neutrophils signify a pro-tumorigenic inflammatory surge, while depleted lymphocytes indicate a compromised adaptive immune response [13,14]. However, the NLR often reflects a generalized, acute-phase stress response that may lack the nuance required to distinguish specific pathologies. Platelet-to-Lymphocyte Ratio (PLR) reflects the relationship between pro-thrombotic inflammation and adaptive immune pathways. High PLR is often associated with a state of heightened systemic inflammation. In oncology, it is frequently utilized as a prognostic marker, where an elevated ratio may correlate with poorer survival outcomes or advanced disease stages [15]. Monocyte-to-Lymphocyte Ratio (MLR) provides additional prognostic depth by reflecting macrophage-driven remodeling [16]; however, these ratios frequently overlook the contribution of eosinophils, which belong to the myeloid (granulocytic) lineage, and, more crucially, the immunomodulatory role of eosinophils in the tumor microenvironment [17].

In endometrial cancer specifically, these CBC-derived indices, together with the systemic immune-inflammation index (SII), have been examined as adjuncts to diagnosis and as prognostic markers; however, the available evidence is heterogeneous and derives largely from neutrophil-, platelet-, and monocyte-based measures, leaving the eosinophil compartment comparatively unexplored [18,19].

Lesser studied are eosinophils; especially their unique role in carcinogenesis [17]. Cancer risk is altered in patients with systemic autoimmune conditions such as SLE, with site-specific patterns, and chronic inflammation has also been linked to reproductive tract pathology; whether chronic pelvic inflammation and eosinophilic activity play specialized roles in reproductive tract defense remains elusive [2,20].

The functional versatility of eosinophils in the tumor microenvironment (TME) presents a complex paradigm. These cells possess cytotoxic granules—Eosinophil Peroxidase (EPO) and Major Basic Protein (MBP)—capable of direct tumor lysis [17]. However, their role is highly plastic; in a Th2-skewed environment, eosinophils may secrete IL-4 and IL-13, promoting M2 macrophage polarization [21], which may in turn dampen anti-tumor immunity. Given this regulatory plasticity, the Eosinophil-to-Lymphocyte Ratio (ELR) is a plausible candidate for reflecting the systemic immune-inflammatory equilibrium.

In gynecological malignancies—specifically cervical, ovarian, and endometrial cancers—reliable, cost-effective, and non-invasive blood-based indices remain elusive [22]. Cervical cancer is the most common gynecological cancer in developing countries; persistent infection with high-risk HPV plays a key role in its pathophysiology [23,24]. Early detection is possible with Pap smears or HPV tests [25]. Ovarian cancer is often diagnosed late because of its insidious onset, and its treatment relies on cytoreductive surgery and platinum-based chemotherapy [26,27]. Uterine cancer, mainly endometrial cancer, is the most common gynecological cancer in developed countries. Type 1 tumors are estrogen-dependent and associated with obesity, insulin resistance, and hyperestrogenism, whereas Type 2 tumors are estrogen-independent and frequently harbor TP53 mutations [28,29]. More recently, The Cancer Genome Atlas proposed a molecular-based classification that shifted the paradigm from histologic to molecular precision in endometrial cancer management [30,31].

The standard marker, CA-125, is notorious for poor specificity, being elevated by benign conditions like pelvic inflammatory disease [32]. Similarly, cytology and HPV co-testing often fail to identify which high-grade squamous lesions will advance. Screening limitations are also relevant to less common cervical histologies: glandular and adenosquamous carcinomas account for a considerable proportion of cervical cancers, are less reliably detected by cytology, and lack dedicated management guidelines [33]. These limitations highlight the need for a dynamic indicator of the host’s “immune tone.” Systemic inflammatory indices, derived from routine blood counts, offer a unique opportunity to monitor the biological ‘soil’ in which tumors grow, providing a more holistic view than single-protein markers [34].

Eosinophilia has also been described in gynecologic tumors at the case level, including an adenosquamous carcinoma of the cervix with tumor-associated tissue eosinophilia and peripheral blood eosinophilia [35], which provides a rationale beyond mechanistic reviews for examining eosinophil-based indices in gynecologic cancers. More broadly, eosinophils in the tumor microenvironment and the ELR itself have been explored as immune-inflammatory indicators in cancer and immune-mediated disease [36,37]. Utilizing comprehensive data from the National Health and Nutrition Examination Survey (NHANES), this research aims to determine whether the ELR is associated with gynecological cancers, with a focus on endometrial cancer, and to explore its potential as a complementary inflammatory marker. To address limitations inherent to single population datasets or small sample sizes, this study adopts a two-stage design: an initial discovery analysis using the NHANES 2017–2023, followed by an analysis of an independent institutional cohort of 901 endometrial cancer cases and 200 benign controls. Because the NHANES outcome is self-reported uterine cancer in a cross-sectional survey, whereas the institutional outcome is histologically confirmed endometrial cancer compared with benign surgical controls, the two analyses differ in outcome definition, comparator population, and covariate adjustment. They are therefore regarded as complementary rather than confirmatory, and the study as a whole is hypothesis-generating.

## 2. Materials and Methods

### 2.1. Study Design and Discovery Cohort (NHANES 2017–2023)

To investigate the association and discriminative capability of a systemic inflammatory marker, a two-stage study design was implemented: a national population-based discovery stage followed by an independent single-center institutional cohort stage.

For the discovery cohort, epidemiological data were extracted from the National Health and Nutrition Examination Survey (NHANES) cycles covering 2017–2020 (pre-COVID-19 pandemic) and 2021–2023 (post-COVID-19 pandemic), conducted by the National Center for Health Statistics (NCHS) of the Centers for Disease Control and Prevention (CDC). NHANES employs a complex, stratified, multistage probability sampling design to obtain a nationally representative sample of the non-institutionalized civilian population of the United States.

### 2.2. Laboratory Assays and Primary Variable Definition

The primary variable of interest in this study was the Eosinophil-to-Lymphocyte Ratio (ELR). Complete blood cell counts (CBCs) with differential leukocyte analyses were performed on whole blood specimens using automated hematology analyzers (e.g., Beckman Coulter DxH 800, Brea, CA, USA) according to standardized, rigorous NCHS laboratory protocols. Full details of the assay methodology, quality control, and laboratory operations are published by the NCHS and are publicly accessible via their official repository. Absolute eosinophil and lymphocyte counts were expressed as 10^3^ cells/uL, and the ELR was calculated as the ratio of absolute eosinophil count to absolute lymphocyte count.

### 2.3. Outcome Definitions and Covariates Selection

Gynecological cancer outcomes in the NHANES discovery cohort were defined based on self-reported health questionnaire responses detailing prior medical history of cervical, uterine, or ovarian cancer. We acknowledge that the NHANES self-reported survey design cannot strictly differentiate histologic subtypes (e.g., endometrial carcinoma vs. uterine sarcoma). Thus, NHANES data represent a generalized population-level discovery cohort for broad uterine malignancies. While each subtype of gynecological malignancy possesses distinct pathophysiological drivers, common baseline covariates were identified and categorized to adjust for potential confounding effects. Covariates included demographic factors (age, race/ethnicity), body mass index (BMI, kg/m^2^), and key metabolic comorbidities, specifically self-reported prior medical histories of hypertension and diabetes mellitus. Serum C-reactive protein (CRP, mg/L) was additionally summarized in the baseline comparisons.

We rigorously debated the inclusion of estrogen exposure-related parameters as potential confounding covariates, such as exogenous hormone exposure (history of oral contraceptive use), parity, and total duration of breastfeeding. However, due to a substantial proportion of missing data and reduced response rates among gynecological cancer participants across survey cycles, including these reproductive variables compromised model stability and reduced the statistical power of the subgroup analyses. Consequently, these reproductive variables were omitted from the final multivariable regression models to preserve model stability; residual confounding by these factors cannot be excluded. The final multivariable model for uterine cancer was restricted to age and BMI, which are established correlates of endometrial cancer, to limit overfitting given the small number of uterine cancer cases. Other potentially relevant covariates, including hypertension, diabetes, and race/ethnicity, were not included in this model, so residual confounding cannot be excluded.

### 2.4. Institutional Clinical Cohort (Clinical Registry)

To directly address the statistical limitations of population survey case counts, explore discriminative performance, and examine the clinical applicability of ELR, an independent hospital-based registry dataset was analyzed. The institutional cohort comprised 901 consecutive patients diagnosed with histopathologically confirmed endometrial cancer (encompassing FIGO Stages I–IV; all were newly diagnosed, treatment-naïve primary cases) and 200 benign surgical control patients who underwent total hysterectomy for non-malignant gynecological conditions at Yonsei University Health System. The recruitment period was between 2021 and 2022, and exclusion criteria as listed below was applied.

To ensure definitive pathologic confirmation of the entire uterus and eliminate diagnostic uncertainty associated with partial sampling (e.g., resectoscopy or polypectomy), only patients who underwent total hysterectomy were included in both groups. Benign hysterectomy patients were chosen as controls because complete pathological examination of the uterus allowed endometrial malignancy to be excluded with certainty; however, these women are not representative of those in whom endometrial cancer would be clinically suspected, which limits the generalizability of the findings (see Discussion).

For the benign control group, indications included benign conditions such as uterine leiomyoma, adenomyosis, and benign endometrial pathology. To strictly eliminate potential premalignant or malignant confounders, patients with pre- or postoperatively identified endometrial intraepithelial neoplasia (EIN), high-grade cervical dysplasia (CIN III or higher), or any suspected malignancy were strictly excluded. Peripheral blood differential counts obtained during the perioperative period were extracted to calculate the preoperative ELR.

To eliminate diagnostic ambiguity and systemic treatment confounders, strict exclusion criteria were applied to both groups:

1. Patients with recurrent endometrial cancer or prior pelvic radiation/systemic chemotherapy;
2. Patients with active systemic infections, documented hematologic disorders, active allergic/atopic conditions (e.g., severe asthma, hypereosinophilic syndrome), active systemic autoimmune diseases, or systemic corticosteroid therapy within 30 days prior to blood sampling;
3. Patients with pre- or postoperatively identified premalignant lesions (e.g., EIN) or other concurrent malignancies.

All routine complete blood counts with leukocyte differentials were collected at initial diagnostic workup within 30 days prior to any surgical intervention or oncologic therapy (median interval between blood sampling and surgery: 12 days, IQR: 7–18 days).

### 2.5. Ethical Considerations and AI Declaration

The discovery phase used primary NHANES data collection, with protocols reviewed and approved by the National Center for Health Statistics (NCHS) Ethics Review Board (ERB) under specific protocol numbers assigned per survey cycle, which are publicly accessible via the official NCHS repository. Written informed consent was obtained from all participants by the NCHS prior to primary survey administration.

For the institutional cohort, the Institutional Review Board (IRB) of Yonsei University Health System approved this study (IRB number: 4-2026-1199; approval date: 18 September 2026) and waived the requirement for informed consent because of the retrospective use of deidentified clinical data.

In accordance with international reporting standards, generative AI tools were used only for the mechanical correction of English grammar, syntax, and spelling. Generative AI was not employed for data analysis, statistical modeling, or original text composition.

### 2.6. Statistical Analysis

To ensure that all discovery cohort estimates were nationally representative of the U.S. civilian population, we strictly adhered to the NHANES analytic guidelines provided by the NCHS. The integration of multiple survey cycles (2017–2020 pre-pandemic and 2021–2023 post-pandemic) necessitated the construction of composite sample weights to account for differing cycle durations and population base totals. Combining data over a cumulative 5.2-year period, the sample weights for the 2017–2020 cycle were adjusted by multiplying the mobile examination center (MEC) weight (WTMECPRP) by 3.2/5.2, while the 2021–2023 cycle weights were adjusted by multiplying the MEC weight (WTMEC2YR) by 2.0/5.2.

In the discovery cohort, baseline characterization of continuous and categorical variables was performed. Bivariate relationships between covariates and primary cancer outcomes were evaluated for statistical significance using weighted Student’s *t*-tests for continuous measures and weighted Chi-square tests for categorical variables. Multivariable survey logistic regression models were subsequently constructed to evaluate the association between ELR and self-reported uterine cancer after adjustment for age and BMI. Analyses were performed on complete cases with available primary variables and covariates. Linear relationships between continuous logit predictors (ELR, age, BMI) and log-odds were assumed for primary modeling. Extreme ELR values were retained to reflect the complete physiological spectrum of circulating leukocyte distributions, and model stability was assessed by examining potential influential observations.

For the institutional cohort, continuous variables were evaluated for distributional normality using the Shapiro–Wilk test. Due to non-normal distributions, continuous parameters (e.g., ELR) were expressed as medians with Interquartile Ranges (IQR) and compared between endometrial cancer cases and benign controls using the non-parametric Mann–Whitney U test (Wilcoxon rank-sum test). Unadjusted binary logistic regression was executed to calculate Odds Ratios (OR) and 95% Confidence Intervals (CI). To enhance clinical interpretability given the small absolute numerical scale of the ratio, ELR was scaled by a factor of 10 to report the OR and 95% CI per 0.1-unit increment in ELR. Because no covariate adjustment was performed in the institutional cohort, these estimates represent crude associations.

To assess discriminative capability, Receiver Operating Characteristic (ROC) curve analysis was conducted. The Area Under the Curve (AUC) was calculated, and an exploratory cut-off value was determined using Youden’s J statistic index, accompanied by corresponding clinical sensitivity and specificity estimates. The cut-off was derived and evaluated in the same sample, and no internal validation (e.g., bootstrap or cross-validation) was performed; the resulting estimates should therefore be interpreted as exploratory.

All statistical computations were executed using R open-source software (version 4.3.1; R Foundation for Statistical Computing, Vienna, Austria). All hypothesis testing was two-tailed, and statistical significance was defined as *p* < 0.05.

## 3. Results

### 3.1. Discovery Cohort Analysis (NHANES 2017–2023)

A total of 8472 participants from the combined NHANES 2017–2023 survey cycles were noted. After exclusion, 6859 participants met the inclusion criteria and were included in the discovery cohort analysis. The sample included 6757 non-cancer control participants and 102 individuals with self-reported gynecological malignancies, including 45 cervical cancer cases, 38 uterine cancer cases, and 19 ovarian cancer cases, as shown in Table 1.

Participants with cervical cancer did not show a significant age difference compared to controls (mean age: 49.86 vs. 47.03 years, *p* = 0.2115) but had a significantly higher prevalence of hypertension (56.48% vs. 29.12%, *p* = 0.0125). There were no statistically significant differences in BMI (30.11 vs. 30.03 kg/m^2^, *p* = 0.9344), serum CRP (6.23 vs. 4.24 mg/L, *p* = 0.3234), or racial distribution (*p* = 0.1045) between the cervical cancer group and controls. The mean ELR in the cervical cancer group (0.0760 ± 0.0053) was numerically lower than that of the control group (0.0868 ± 0.0019), but this difference was not statistically significant (*p* = 0.4763).

The uterine cancer group was significantly older than the control group (mean age: 66.23 ± 1.28 vs. 47.03 ± 0.57 years, *p* < 0.001). This group also exhibited a significantly higher prevalence of hypertension (57.93% vs. 29.12%, *p* = 0.0133) and a significantly different racial distribution (*p* = 0.0185), characterized by a higher proportion of Non-Hispanic Whites (81.47%) compared to the control cohort (58.71%). Notably, the mean ELR was significantly elevated in the uterine cancer group compared to controls (0.1204 ± 0.1090 vs. 0.0868 ± 0.0019, *p* = 0.0193). While BMI (33.95 ± 2.88 vs. 30.03 ± 0.26 kg/m^2^, *p* = 0.1136) and diabetes prevalence (17.52% vs. 9.55%) were numerically higher in the uterine cancer group, these differences did not reach statistical significance (*p* = 0.4363).

The ovarian cancer group was also significantly older than controls (mean age: 59.88 ± 2.57 vs. 47.03 ± 0.57 years, *p* < 0.001). There were no statistically significant differences in race, BMI, hypertension, diabetes, or serum CRP between the ovarian cancer group and controls (all *p* > 0.05). Although the mean ELR (0.1015 ± 0.0233) was numerically higher than that of the control group (0.0868 ± 0.0019), the difference did not achieve statistical significance (*p* = 0.4598).

In the multivariable survey logistic regression for uterine cancer (adjusted for age and BMI; Table 2), ELR was associated with higher odds of self-reported uterine cancer (OR = 10.288 per 1.0-unit increase, 95% CI: 1.722–61.472, *p* = 0.0130; equivalent to OR = 1.26 per 0.1-unit increase, 95% CI: 1.06–1.51); however, the wide confidence interval reflects the small number of cases (*n* = 38), and the estimate may be unstable. In the cervical cancer model, ELR showed a non-significant inverse association (OR = 0.022 per 1.0-unit increase, 95% CI: 0.0004–1.174, *p* = 0.0591; OR = 0.68 per 0.1-unit increase, 95% CI: 0.46–1.02), whereas in the ovarian cancer model, age was the only significant factor (OR = 1.043 per year, 95% CI: 1.017–1.070, *p* = 0.0019). The covariates entered into the subtype-specific models differed, as shown in Table 2.

### 3.2. Independent Institutional Cohort

To examine whether the direction of the NHANES finding was also observed in a histologically confirmed endometrial cancer population and to explore the discriminative performance of ELR, a total of 1101 participants (901 patients with histopathologically confirmed endometrial cancer and 200 benign surgical controls) were analyzed.

In non-parametric baseline comparison, the median pre-treatment ELR was significantly higher in endometrial cancer patients than in benign surgical controls (0.1180 [IQR: 0.1380] vs. 0.0487 [IQR: 0.0574], *p* < 0.001 by Mann–Whitney U test).

In unadjusted binary logistic regression scaled to assess the odds per 0.1-unit increment, a higher ELR was significantly associated with higher odds of endometrial cancer (OR = 2.725, 95% CI: 2.154–3.515, *p* < 0.001).

**Figure 1:**
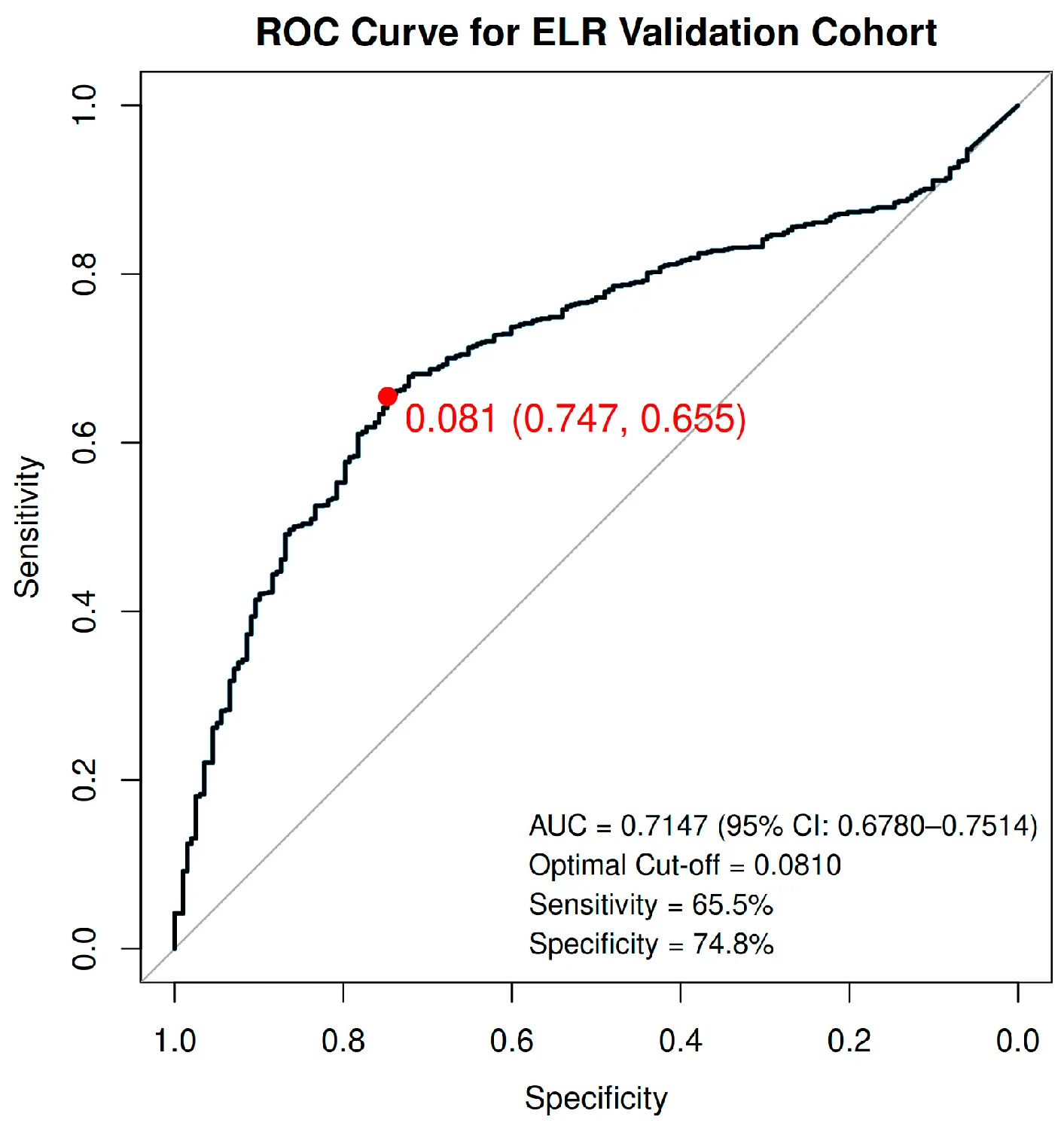
Receiver Operating Characteristic (ROC) curve analysis of the Eosinophil-to-Lymphocyte Ratio (ELR) in the independent institutional cohort (N = 1101). The blue solid line represents the discriminative performance of preoperative ELR for distinguishing endometrial cancer cases (N = 901) from benign surgical controls (N = 200), yielding an Area Under the Curve (AUC) of 0.7147 (95% CI: 0.6780–0.7514). The exploratory cut-off value calculated via Youden’s J index was 0.0810, with a corresponding sensitivity of 65.5% and specificity of 74.8%.

As shown in Figure 1, Receiver Operating Characteristic (ROC) curve analysis demonstrated moderate discriminative performance for pre-treatment ELR, yielding an Area Under the Curve (AUC) of 0.7147 (95% CI: 0.6780–0.7514). According to Youden’s J statistic, an exploratory cut-off value for ELR was 0.0810, achieving a clinical sensitivity of 65.5% and a specificity of 74.8%. These estimates were derived and evaluated in the same sample and were obtained in comparison with benign surgical controls; they should not be interpreted as applicable to screening or routine diagnostic triage.

## 4. Discussion

This study evaluated the Eosinophil-to-Lymphocyte Ratio (ELR) as a biomarker associated with endometrial cancer through a two-stage approach combining a national epidemiological discovery cohort (NHANES 2017–2023) with an independent institutional cohort (*n* = 1101). In the discovery cohort, multivariable logistic regression showed that a higher ELR was associated with self-reported uterine cancer (OR = 10.288 per 1.0-unit increase, equivalent to 1.26 per 0.1-unit increase; *p* = 0.0130) after adjusting for age and BMI, although the small number of cases (*n* = 38) yielded a wide confidence interval. In the institutional cohort, higher ELR was likewise associated with endometrial cancer in unadjusted analysis (OR = 2.725 per 0.1-unit increase, 95% CI: 2.154–3.515, *p* < 0.001) and showed moderate discriminative performance (AUC = 0.7147) with an exploratory threshold of 0.0810 (sensitivity: 65.5%, specificity: 74.8%). Although the two cohorts differ in outcome definition and comparator group, the direction of association was consistent, suggesting that ELR is a candidate low-cost, non-invasive inflammatory index that warrants further evaluation. These findings should be regarded as hypothesis-generating: the NHANES analysis concerns prevalent, self-reported uterine cancer in a cross-sectional design, the institutional analysis concerns histologically confirmed endometrial cancer without confounder adjustment, and evidence for an independent association after adequate adjustment is therefore limited.

The biological mechanisms linking systemic eosinophil shifts to endometrial malignancy remain speculative within the context of our study design. The prior literature suggests that eosinophils show functional plasticity and a dual, context-dependent role in the tumor microenvironment [36,38,39,40], and that estrogen signaling may promote eosinophil recruitment and Th2-skewed cytokine release [39,41]. However, estrogen levels, tumor eosinophil infiltration, eotaxin expression, and relevant cytokines were not measured in this study. Any proposed link between a hormone-driven eosinophil response and uterine cancer should therefore be regarded as a hypothesis only, and translational tissue-level studies are needed to test whether systemic ELR reflects local eosinophil trafficking.

A notable feature of our analysis is the use of unit-scaled odds ratios to aid interpretation. Because ELR values lie on a small decimal scale (typically between 0.04 and 0.15), odds ratios per 1.0-unit increase (e.g., OR = 10.288 in NHANES) are numerically large and difficult to interpret clinically. We therefore also expressed effect sizes per 0.1-unit increase (OR = 1.26 in NHANES; OR = 2.725 in the institutional cohort); in the institutional cohort, each 0.1-unit higher preoperative ELR was associated with 2.725-fold higher unadjusted odds of endometrial cancer (a 172.5% increase). It is important to note that because these odds ratios derive from different comparison groups, outcome definitions, and adjustment sets, they should not be compared directly across cohorts. Furthermore, our findings do not establish ELR as a standalone screening or confirmative diagnostic tool for population-wide early detection. Rather, given its moderate discriminative capacity (AUC = 0.7147), ELR may represent a low-cost, readily available complementary biomarker that could be evaluated together with standard clinical evaluation and ultrasound imaging; this combination could serve as a future area of investigation.

The association in NHANES persisted after adjustment for age and BMI; however, hypertension, diabetes, and other metabolic factors were not included in that model and the institutional analysis was unadjusted, so we cannot conclude that ELR is independent of metabolic syndrome or obesity-related inflammation.

This focus on leukocyte-derived ratios aligns with the growing body of evidence regarding the systemic inflammatory response in gynecological cancers. Cho et al. previously demonstrated the clinical utility of the Neutrophil-to-Lymphocyte Ratio (NLR) as a discriminative biomarker, showing that elevated NLR was the most powerful predictive variable for adverse outcomes in epithelial ovarian cancer [14]. While their study highlighted the prognostic value of neutrophils in ovarian cancer, our research explores eosinophils as a possible, but unproven, component of the inflammatory profile of uterine cancer. Whereas Cho et al. reported elevated NLR in ovarian cancer, our data suggest an association between ELR and uterine cancer; however, because we did not directly compare ELR with NLR, PLR, MLR, or other inflammatory indices in the institutional cohort, we cannot definitively conclude that ELR has greater discriminative value than these markers.

The divergent findings across cancer types also merit attention. In contrast to uterine cancer, cervical cancer showed a non-significant, borderline inverse association with ELR (OR = 0.022 per 1.0-unit increase, *p* = 0.0591), based on only 45 cases and with an extremely wide confidence interval (0.0004–1.174) that included the null. This result is imprecise and should be regarded with caution; it does not allow conclusions about a protective role of eosinophils in the cervical tumor microenvironment, and the covariate set of the cervical model differed from that of the uterine model. Whether the interpretation of ELR is cancer-specific needs to be tested in future studies.

Furthermore, the persistence of the ELR association after adjustment for age and BMI in NHANES is of interest. In uterine cancer, where obesity is a major confounding factor, a biomarker that retains information after accounting for BMI could, if confirmed, merit further evaluation as a complementary marker.

Beyond the biological implications, survey-weighted analysis of the NHANES 2017–2023 dataset allowed us to account for the complex survey design, which improves the national representativeness of the discovery-stage estimates for the non-institutionalized U.S. civilian population. However, survey weighting does not remove selection bias, misclassification of self-reported outcomes, or confounding, and the estimate for uterine cancer (OR = 10.288 per 1.0-unit increase) is imprecise owing to the small number of cases. Whether ELR carries information beyond obesity-related low-grade inflammation, or compares favorably with standard inflammatory markers such as CRP, was not assessed and requires dedicated comparison.

Several limitations should be noted. First, the NHANES discovery dataset relied on self-reported questionnaire data, which cannot distinguish endometrial carcinoma from other uterine neoplasms such as uterine sarcoma. ELR was measured after the reported diagnosis in a cross-sectional design, so temporality and causality cannot be inferred, and the odds ratios reflect associations with prevalent self-reported cancer rather than risk. The number of cancer cases was small (38 uterine, 45 cervical, and 19 ovarian), producing wide confidence intervals; three cancer subtypes were examined without adjustment for multiple comparisons; the covariate sets differed among subtype-specific models; and the uterine cancer model did not include hypertension or diabetes. Residual confounding by race/ethnicity, reproductive factors, and hormonal exposures also cannot be excluded, and with only a small number of uterine cancer cases, model stability and possible overfitting remain concerns. Accordingly, the findings should not be interpreted as evidence that ELR predicts future cancer risk.

Second, the institutional cohort represents a clinical analysis in a histologically confirmed endometrial cancer population rather than an identical epidemiological duplicate. Its logistic regression was unadjusted, so age, BMI, menopausal status, and metabolic comorbidities could have served as confounding factors; in particular, women undergoing hysterectomy for benign conditions are typically younger than those with endometrial cancer. The use of benign surgical controls (case-to-control ratio of approximately 4.5:1) may introduce spectrum bias, so the sensitivity, specificity, and cut-off value are not directly transferable to screening populations. Differences between cases and controls in menopausal status, obesity, inflammatory conditions, and surgical indications may also influence leukocyte distributions, and baseline demographic and clinical characteristics of the institutional cohort were not analyzed or adjusted for in this study; consequently, the association observed in this cohort cannot be considered independent, and the terms diagnostic threshold and diagnostic performance should be interpreted cautiously. Furthermore, ELR was not compared with a clinical model including established predictors such as age and BMI, so its incremental value was not assessed. In addition, the cut-off value was derived and evaluated in the same sample without internal validation, which may overestimate performance.

Third, although the institutional cohort was restricted to treatment-naïve primary cases, unmeasured clinical confounders such as menopausal status or specific hormone replacement therapy were not fully captured, and ELR is a ratio whose elevation may reflect higher eosinophil counts, lower lymphocyte counts, or both; these components were not analyzed separately, which limits the biological interpretation of the ELR finding and of the mechanistic hypotheses discussed above. Fourth, while strict clinical exclusion criteria were applied regarding steroid use and systemic inflammation, minor uncaptured atopic conditions could subtly influence circulating leukocyte counts. In addition, ELR was not compared with other CBC-derived indices (NLR, PLR, MLR), so any advantage over established indices remains untested. Finally, prospective longitudinal studies are required to confirm these findings in adjusted models, to track dynamic ELR shifts during treatment, and to evaluate its prognostic value for long-term oncologic outcomes.

Despite these limitations, the consistent direction of association observed across national population survey data and a large institutional cohort supports further evaluation of ELR. Given that complete blood counts are routinely performed in standard gynecological care, calculating ELR requires no additional healthcare cost or specialized laboratory testing. As a ratio derived from a standard CBC, ELR could, if validated, be a cost-effective and readily available index in primary care and specialized oncology settings. Future studies should evaluate whether incorporating ELR alongside established clinical evaluation and other markers adds information to the evaluation of endometrial cancer, which was not examined here. This study illustrates how public data from NHANES can be used to generate hypotheses about low-cost biomarkers that can then be tested in clinical cohorts.

## 5. Conclusions

In NHANES, ELR was higher in women with self-reported uterine cancer than in non-cancer controls, and the association persisted after adjustment for age and BMI. In the institutional cohort, ELR was higher in patients with endometrial cancer than in benign surgical controls (unadjusted analysis). With an exploratory cut-off of 0.0810 and an AUC of 0.7147, ELR showed moderate discriminative performance in this selected comparison. These hypothesis-generating findings do not establish an independent association; ELR may be a candidate accessible, non-invasive complementary marker, but its clinical utility requires confirmation in adjusted, prospective studies that also compare it with established inflammatory indices.

## Figures and Tables

**Table 1:** Demographic characteristics of the NHANES population from 2021 to 2023.

	Control Mean (SE)	Cervical Cancer		Uterine Cancer		Ovarian Cancer	
		Mean (SE)	*p*-Value	Mean (SE)	*p*-Value	Mean (SE)	*p*-Value
Age	47.03 (0.57)	49.86 (2.00)	0.2115	66.23 (1.28)	<0.001	59.88 (2.57)	<0.001
Race/ethnicity			0.1045		0.0185		0.6830
Mexican American	7.97% (1.03)	2.28% (1.48)		2.32% (1.50)		3.00% (2.97)	
Other Hispanic	9.16% (0.78)	4.27% (1.77)		3.16% (1.97)		12.54% (7.03)	
Non-Hispanic White	58.71% (1.74)	74.98% (6.96)		81.47% (6.88)		65.17% (14.58)	
Non-Hispanic Black	12.88% (1.19)	8.72% (3.57)		5.71% (3.07)		5.58% (3.65)	
Other	11.28% (0.95)	9.75% (3.08)		7.34% (4.31)		13.70% (10.46)	
BMI	30.03 (0.26)	30.11 (1.66)	0.9344	33.95 (2.88)	0.1136	29.68 (3.55)	0.8718
Hypertension	29.12% (0.83)	56.48% (10.03)	0.0125	57.93% (11.83)	0.0133	56.62% (0.18)	0.1113
Diabetes	9.55% (0.55)	9.82% (4.01)	0.9295	17.52% (7.29)	0.4363	22.20% (11.86)	0.5408
ELR	0.0868 (0.0019)	0.0760 (0.0053)	0.4763	0.1204 (0.109)	0.0193	0.1015 (0.0233)	0.4598
CRP	4.24 (0.22)	6.23 (1.97)	0.3234	5.69 (1.19)	0.2478	3.58 (1.24)	0.5669

Note: Data are presented as weighted means with standard errors (SE) for continuous variables and percentages (%) for categorical variables. *p*-values were derived from weighted Student’s *t*-tests for continuous measures and weighted Chi-square tests for categorical variables.

**Table 2:** Multivariable Survey Logistic Regression Analyses in the NHANES Cohort.

Cancer Subtype	Parameter	Beta	SE beta	OR	OR 95 CI	*p*-Value
Cervical cancer	ELR	−3.8270	1.9225	0.0218	0.0004–1.1736	0.0591
	Hypertension	−1.0907	0.4540	0.3360	0.1310–0.8615	0.0251
	CRP	0.0102	0.0079	1.0103	0.9938–1.0271	0.2110
Uterine cancer	ELR	2.3309	0.8620	10.2876	1.7216–61.4720	0.0130
	Age	0.0804	0.0100	1.0837	1.065–1.1065	<0.001
	BMI	0.0564	0.0317	1.0580	0.9907–1.1299	0.0891
Ovarian cancer	Age	0.0422	0.0121	1.0431	1.0174–1.0695	0.0019
	DM	−0.7240	0.6430	0.4848	0.1282–1.8333	0.2717

Note: SE = Standard Error; CI = Confidence Interval. All models were adjusted for complex survey sample design weights.

## Data Availability

Publicly available datasets were analyzed in this study. This data can be found here: https://www.cdc.gov/nchs/nhanes/about-data/index.html (accessed on 30 December 2025). The institutional dataset contains patient-level clinical data and is not publicly available because of patient privacy and institutional restrictions.

## Abbreviations

The following abbreviations are used in this manuscript:

NHANES	National Health and Nutrition Examination Survey
NCHS	National Center for Health Statistics
DM	Diabetes mellitus
BMI	Body mass index
OR	Odds ratio
CI	Confidence interval
SE	Standard error
CRP	C-Reactive Protein
ELR	Eosinophil-to-Lymphocyte Ratio
NLR	Neutrophil-to-Lymphocyte Ratio
PLR	Platelet-to-Lymphocyte Ratio
MLR	Monocyte-to-Lymphocyte Ratio
SII	Systemic immune-inflammation index
WBC	White blood cell
ROS	Reactive Oxygen Species
RNS	Reactive Nitrogen Species
VEGF	Vascular Endothelial Growth Factor
EPO	Eosinophil Peroxidase
MBP	Major Basic Protein
TME	Tumor Microenvironment
ICIs	Immune Checkpoint Inhibitors
HPV	Human Papillomavirus
SLE	Systemic Lupus Erythematosus
IRB	Institutional Review Board
